# High-intensity ultrasound-assisted recovery of type II collagen from porcine trachea: Enhanced recovery and multiscale structural organization

**DOI:** 10.1016/j.ultsonch.2026.107973

**Published:** 2026-07-21

**Authors:** Kitsanapong Kaewbangkerd, Ekkasit Kanklang, Apichart Ngernsoungnern, Catleya Rojviriya, Sukanya Tastub, Sittichoke Sinthusamran, Jirawat Yongsawatdigul

**Affiliations:** a School of Food Technology, Institute of Agricultural Technology, Suranaree University of Technology, Nakhon Ratchasima 30000, Thailand; b School of Pre-clinic, Institute of Science, Suranaree University of Technology, Nakhon Ratchasima 30000, Thailand; c Synchrotron Light Research Institute, Nakhon Ratchasima 30000, Thailand; d CPF Food Research and Development Center, CPF (Thailand) Public Company Limited, Ayutthaya 13170, Thailand

**Keywords:** Porcine trachea, Type II collagen, High-intensity ultrasound, Cartilage matrix, Synchrotron tomography

## Abstract

Porcine trachea is an underutilized co-product with high potential as a source of type II collagen (CII). This study evaluated high-intensity ultrasound (HIU) as a pretreatment to enhance collagen extraction efficiency while preserving structural integrity. Collagen obtained by acid (PTC-A), pepsin (PTC-P), and ultrasound–pepsin treatment (PTC-U) was compared. Under optimized conditions (23.75 W/cm^2^, 15 min), HIU increased collagen yield to 19.97 % and reduced extraction time by approximately 58 % compared to conventional methods. Acid extraction (PTC-A) yielded only 1.82 % and predominantly recovered type I collagen, whereas pepsin-assisted extraction (PTC-P) achieved 19.91 % and enabled enrichment of cartilage-derived CII. The combined treatment (PTC-U) further improved collagen content to 80.51 % (vs. 75.04 % for PTC-P). Amino acid analysis confirmed a typical CII profile, with glycine (173–208 residues/1,000 residues) and high imino acid content (proline + hydroxyproline: ∼270 residues/1,000 residues), supporting triple-helix stability. Scanning electron microscopy and synchrotron radiation X-ray tomographic microscopy revealed a compact lamellar (layer-like) three-dimensional architecture with reduced porosity (77.97 ± 2.75 %), compared to PTC-A (85.70 ± 0.29 %) and PTC-P (81.82 ± 0.30 %). Circular dichroism, Fourier transform infrared spectroscopy, and synchrotron radiation wide-angle X-ray scattering confirmed preservation of the native triple-helical structure. All samples exhibited comparable denaturation temperatures (∼39.7–39.9 °C) based on micro-differential scanning calorimetry. Proteomic analysis further verified that acid extraction yielded type I collagen, whereas HIU-assisted extraction enriched type II collagen.

## 1 Introduction

Global porcine slaughterhouse operations generate substantial quantities of underutilized co-products, including respiratory tissues such as the trachea [1]. The porcine trachea is structurally composed of connective tissue, smooth muscle, and C-shaped hyaline cartilage [2], which is particularly rich in type II collagen (CII), accounting for approximately 95 % of total collagen in vertebrate cartilage [3]. Utilization of porcine trachea for collagen recovery represents a value-added approach that may improve resource utilization and reduce waste associated with animal processing. Consequently, the objective of collagen extraction from cartilage is not simply to maximize collagen recovery, but to selectively recover type II collagen while preserving its native molecular structure.

Type II collagen is structurally distinct from type I collagen, consisting of a homotrimeric assembly of three identical α1(II) chains that form a stable triple-helical conformation [4], [5]. In native cartilage, CII is embedded within a highly organized extracellular matrix composed of proteoglycans and other macromolecules, forming a dense and complex network [6], [7]. This structural organization presents a significant barrier to efficient extraction, as extensive intermolecular crosslinking and matrix entrapment limit collagen solubilization [8]. Consequently, recovery of undenatured CII remains technically challenging, despite its increasing importance in biomaterials, nutraceuticals, and functional biopolymer applications [7], [9], [10], [11].

Conventional collagen extraction methods, including acid solubilization and pepsin-assisted hydrolysis, have been widely applied to various cartilaginous tissues [12], [13]. While acid treatment disrupts intermolecular interactions, it is often insufficient for highly cross-linked cartilage matrices [10]. Pepsin improves extraction efficiency by selectively cleaving non-helical telopeptide regions, thereby increasing collagen solubility without damaging the triple helix [10], [13]. However, these approaches typically require prolonged processing times and still suffer from limited yield and scalability [13], [14]. These limitations highlight the need for innovative extraction strategies capable of enhancing mass transfer and enzyme accessibility without compromising structural integrity [11], [15].

High-intensity ultrasound (HIU) has emerged as a promising green technology for improving extraction efficiency in biopolymer processing. The propagation of ultrasound waves in liquid media generates acoustic cavitation, leading to the formation and collapse of microbubbles that produce localized shear forces, microjetting, and transient high-energy conditions [15], [16]. These effects can disrupt biological matrices, reduce diffusion barriers, and enhance solvent and enzyme penetration [15]. Previous studies have demonstrated that ultrasound pretreatment can significantly increase collagen yield from complex biological tissues such as chicken sternum and ovine bone [17], [18]. However, excessive ultrasound intensity or prolonged exposure may induce partial denaturation of the triple-helix structure, thereby compromising functional properties. Therefore, achieving a balance between matrix disruption and structural preservation remains a critical challenge in ultrasound-assisted collagen extraction [17], [19], [20].

Although ultrasound-assisted extraction has previously been applied to collagen recovery from connective tissues and selected cartilage sources [11], [17], previous studies including our earlier work on broiler chicken trachea [21] primarily focused on improving collagen extraction efficiency without addressing the selective recovery of cartilage-specific type II collagen. Whether HIU can facilitate extraction of type II collagen from the dense proteoglycan-rich matrix of porcine trachea while preserving its molecular organization remains unknown.

Therefore, this study aimed to establish porcine trachea as a novel source of type II collagen and to determine whether controlled HIU pretreatment can selectively enhance recovery of cartilage-derived type II collagen while preserving its triple-helical structure. Unlike our previous work that focused on collagen extraction from chicken trachea [21], the present study addresses the fundamentally different challenge of recovering cartilage-specific type II collagen from a highly organized proteoglycan-rich extracellular matrix. Collagen obtained via ultrasound-assisted extraction was systematically compared with acid- and pepsin-solubilized counterparts in terms of yield, biochemical composition, and structural characteristics. Advanced analytical techniques, including synchrotron-based imaging and spectroscopy, were employed to provide detailed insights into collagen organization and stability.

## 2 Materials & methods

### 2.1 Chemicals, reagents, and raw materials

Pepsin from porcine gastric mucosa (EC 3.4.23.1), *trans*-4-hydroxy-L-proline, type II collagen (chicken sternum), type I collagen (calf skin), gelatin (porcine skin), chloramine-T hydrate, 4-(dimethylamino) benzaldehyde (p-DMAB), β-mercaptoethanol (BME), and Bouin’s fixative were purchased from Sigma-Aldrich (St. Louis, MO, USA). Alcian blue 8GN, alizarin red S, and trypsin from bovine pancreas (EC 3.4.21.4) were purchased from HiMedia Laboratories (Maharashtra, India). A type II collagen enzyme-linked immunosorbent assay (ELISA) kit was purchased from Chondrex Inc. (Woodinville, WA, USA). Bicinchoninic acid (BCA) protein assay kits were purchased from Merck Millipore (Burlington, MA, USA). Ninhydrin was purchased from Biochrom Ltd. (Cambridge, UK). Molecular weight ladders of 180–11 kilodaltons (kDa) and Coomassie nano protein staining solution were purchased from Bio-Helix Co., Ltd. (New Taipei, Taiwan). All electrophoresis-grade chemicals were obtained from Bio-Rad Laboratories (Hercules, CA, USA), and other analytical-grade reagents were purchased from PanReac AppliChem (Darmstadt, Germany).

Fresh porcine trachea samples were obtained from Charoen Pokphand Foods (Saraburi, Thailand) a licensed commercial slaughterhouse (Charoen Pokphand Foods, Saraburi, Thailand) operating under national food safety regulations and animal welfare standards, including humane handling, transportation, pre-slaughter management, and stunning procedures designed to minimize animal stress and suffering. Importantly, the animals were not slaughtered specifically for collagen extraction or research purposes; rather, the tracheae were collected as slaughterhouse by-products that would otherwise have limited value. Samples were transported under chilled conditions (4 °C) to Suranaree University of Technology (Nakhon Ratchasima, Thailand) within 2 h.

### 2.2 Proximate analysis

The ash, protein, moisture, and hydroxyproline (Hyp) contents were determined according to the AOAC international methods [20], as described in method No. 920.153, 928.08, 950.46, and 990.26, respectively. A conversion factor of 6.25 was used to calculate crude protein content. For the Hyp assay, all samples were subjected to alkaline hydrolysis in an autoclave at 121 °C for 40 min. Free Hyp was then reacted with chloramine-T and Ehrlich's aldehyde. The absorbance of the sample solution (λ_max_) was measured at 550 nm [22]. The total collagen content was evaluated by multiplying the Hyp content by 7.6 as a conversion factor [21]. Total lipids were analyzed using chloroform: methanol extraction according to Folch et al. [23].

### 2.3 Porcine tracheal microanatomy

#### 2.3.1 Histology and histochemical analysis

Tracheal rings were collected and immediately fixed in Bouin’s solution. Samples were dehydrated through a graded ethanol series (70–100 %, v/v), embedded in paraffin, and sectioned at 5 µm using a rotary microtome (HistoCore BIOCUT, Leica Microsystems, USA). Tissue sections were stained with hematoxylin and eosin (H&E) to assess general histological features. Type I collagen distribution was evaluated using Picro-Sirius Red staining to differentiate the surrounding fibrous connective tissue from the hyaline cartilage. Images were captured using a bright-field microscope (Zeiss GmbH, Germany) at magnifications of 4×, 20×, and 40×.

#### 2.3.2 Diaphonization

Whole tracheal samples were processed following a modified diaphonization protocol [24]. Specimens were fixed in 10 % (v/v) formalin for 24 h, rinsed with deionized water, and stained with 0.1 % (w/v) Alcian blue (prepared in acetic acid:ethanol, 1:2, v/v) for 24 h to visualize cartilage components. Samples were rehydrated through decreasing ethanol concentrations (95–25 %, v/v) and incubated in water prior to enzymatic digestion using trypsin solution (0.5 g in 30 % sodium tetraborate) for 72 h to remove soft tissues.

Calcified structures were subsequently stained with 0.2 % (w/v) Alizarin red S in 0.5 % (w/v) KOH for 24 h. Specimens were then cleared through graded glycerol/KOH solutions (3:1, 1:1, 1:3, v/v) and stored in glycerol containing 0.05 % (w/v) thymol for preservation.

### 2.4 Protein extraction and analysis

#### 2.4.1 Sample preparation

Connective tissue (CT) and cartilage rings (CR) were manually separated from whole trachea (T). The CR fraction was pretreated with 0.1 M NaOH at a solid-to-liquid ratio of 1:10 (w/v) for 5 days, with daily replacement of the alkali solution to remove non-collagenous proteins [13], [25]. T, CT, and pretreated CR samples were then individually blended and further homogenized using a high-speed disperser (Ultra-Turrax T25, IKA, Germany) in extraction buffer containing 8 M urea, 10 % (w/v) SDS, and 2 % (v/v) BME. The homogenates were centrifuged (10,000 × g, 10 min), and the supernatants were collected and dialyzed against 0.1 % (w/v) SDS for 24 h prior to analysis.

#### 2.4.2 Protein patterns (SDS-PAGE)

Protein profiles were analyzed by SDS-PAGE [26]. Dialyzed samples were mixed with sample buffer (0.5 M Tris-HCl, pH 6.8, containing 4 % SDS, 10 % BME, 20 % glycerol, and 0.02 % bromophenol blue) and adjusted to 20 µg protein per lane, as determined by the BCA assay. The mixtures were heated at 95 °C for 5 min prior to loading. Samples were separated on 10 % polyacrylamide gels at a constant voltage of 80 V. Protein bands were visualized using Coomassie Brilliant Blue staining and imaged with a ChemiDoc™ MP system (Bio-Rad, USA). Band molecular weight and intensity were analyzed using UV1D software (Uvitec, UK).

#### 2.4.3 Type II collagen quantification

Type II collagen content in dialyzed fractions was quantified using the Chondrex ELISA kit following the manufacturer’s instructions. Briefly, samples were analyzed using a sandwich ELISA format on 96-well plates, and absorbance was measured at 490 nm using a microplate reader (Varioskan Lux, Thermo Fisher Scientific, Finland). Collagen content was calculated from a standard calibration curve.

### 2.5 Collagen extraction process

#### 2.5.1 Conventional extraction

Alkali pretreatment of porcine trachea (T) was performed according to Pan et al. [25] with slight modification. The pretreated samples were suspended in 0.5 M acetic acid at a solid-to-liquid ratio of 1:15 (w/v) and extracted for 24 h at 25 ± 2 °C under continuous stirring. The mixture was centrifuged (15,000 × g, 15 min), and collagen in the supernatant was precipitated by adding NaCl to a final concentration of 2.5 M. The precipitate was collected by filtration through double-layer cheesecloth, desalted by diafiltration using a 3.5 kDa molecular weight cut-off membrane against deionized water at 4 ± 2 °C for 48 h, and subsequently freeze-dried. The resulting collagen was designated as acid-soluble porcine tracheal collagen (PTC-A).

The residual pellet was further subjected to pepsin-assisted extraction in 0.5 M acetic acid containing pepsin (2,000 units/g residue) at a ratio of 1:15 (w/v) for 12 h at 25 ± 2 °C. Solubilized collagen was recovered using the same precipitation and purification procedures and designated as pepsin-solubilized collagen (PTC-P). Collagen yield and recovery were calculated on a dry basis as follows:

$$Yield\;mass\;\left(\%\right)\;=\;\left(\frac{Weight\;of\;lyophilized\;collagen\;extract\;}{Dry\;weight\;of\;porcine\;trachea}\right)\times 100$$

$$Collagen\;recovery\;\left(\%\right)\;=\;\left(\frac{Collagen\;content\;of\;lyophilized\;powder}{Collagen\;content\;of\;porcine\;trachea}\right)\times 100$$

#### 2.5.2 High-intensity ultrasound (HIU) -assisted extraction

Ultrasound pretreatment was performed using a probe-type ultrasonic processor (Qsonica LLC, USA) operating at a fixed frequency of 20 kHz. Alkali-pretreated tracheal samples (80 g) were suspended in 400 mL of deionized water with the addition of ice to maintain low temperature during sonication. Treatments were conducted in a jacketed glass vessel using a 25 mm titanium probe immersed approximately 5 cm below the liquid surface. Temperature was monitored throughout all HIU treatments using a calibrated thermocouple inserted at mid-vessel depth, with readings recorded at 5 min intervals. Under the selected condition (23.75 W/cm^2^, 15 min, pulsed), the medium temperature remained below 15 °C, with a maximum recorded value of 12.3 ± 1.1 °C.

Ultrasonication was applied in pulsed mode (5 s on/5 s off) at acoustic intensities of 9.18, 15.43, and 23.75 W/cm^2^ for 10, 15, or 20 min. A separate aliquot of ultrasound-treated tracheal tissue was thoroughly rinsed with excess deionized water to remove potential metal residues and subsequently subjected directly to pepsin-assisted extraction in 0.5 M acetic acid under the same conditions described in Section 2.5.1, without a preceding acid extraction step. The extraction time ranged from 2 to 12 h. This sequential, decoupled design allows the effects of HIU (matrix disruption) to be distinguished from those of pepsin (enzymatic digestion). Collagen recovery was evaluated to determine most effective condition among those tested based on yield, recovery, and collagen content. Collagen obtained under the best performing HIU-assisted conditions was designated as PTC-U.

To determine the acoustic intensity, the actual ultrasonic power (W) dissipated into the medium was evaluated calorimetrically by recording the temperature rise during the first 180 s of sonication without cooling. The power was calculated using the equation P = m⋅C_p_⋅(dT/dt), where m is the mass of the solvent (g), C_p_ is the specific heat capacity of water (4.18 J/g^o^C), and dT/dt is the initial rate of temperature rise. The acoustic intensity (W/cm^2^) was then calculated by dividing the power by the surface area of the emitting probe tip, following established calorimetric approaches [15], [27].

### 2.6 Structural and biochemical characteristics

#### 2.6.1 Microstructure

##### 2.6.1.1 Scanning electron microscopy (SEM)

All lyophilized collagen extracts were examined using SEM (Quanta 450, Field Electron and Ion Co., Hillsboro, OR, USA). The specimens were mounted on an aluminum stub holder with carbon adhesive tape and coated with gold ions using an ion beam sputter (JFC-1100E, Japan Electron Optics Laboratory Technics Co. Ltd., Tokyo, Japan). Microstructural observations were performed at an accelerating voltage (HV) of 20 kV with the magnification of 1,000×.

##### 2.6.1.2 Synchrotron radiation X-ray tomographic microscopy (SR-XTM)

The three-dimensional (3D) microstructure of lyophilized collagen samples was analyzed using synchrotron radiation X-ray tomographic microscopy at beamline BL1.2 W, Synchrotron Light Research Institute (SLRI, Thailand). The X-ray source was generated by a 2.4 T multipole wiggler operating at an electron energy of 1.2 GeV, producing filtered polychromatic X-rays with an effective energy of 11.5 keV. Projection images were acquired over a rotation range of 0–180° with an angular step of 0.1°, using a detection system comprising a YAG:Ce scintillator, a lens-coupled X-ray microscope, and an sCMOS camera (PCO.edge 5.5, 2,560 × 2,160 pixels, 16-bit). Raw projections were corrected using flat-field normalization and reconstructed into cross-sectional images via a filtered back-projection algorithm implemented in Octopus Reconstruction software (v8.9.4, TESCAN-XRE, Belgium). The reconstructed volumes were visualized using Drishti software, and quantitative porosity was determined using Octopus Analysis (v8.9.4). This approach enabled high-resolution evaluation of internal structural organization and porosity of collagen matrices. All collagen samples (PTC-A, PTC-P, and PTC-U) were lyophilised using the same freeze-dryer, identical cycle parameters (shelf temperature − 50 °C, primary drying at 0.05 mbar), and were stored at − 20 °C until further use, ensuring that observed differences in porosity reflect intrinsic structural differences rather than processing artefacts.

#### 2.6.2 Amino acid profiles

The amino acid composition was evaluated according to the official AOAC method [20], as described in method no. 2,012.07. Collagen samples were hydrolyzed with 6 M HCl containing 0.1 % (w/v) phenol at 115 °C for 24 h. Norleucine was added to the samples as an internal standard. Amino acid content was quantified using an amino acid analyzer (Biochrom 30 plus, Biochrom Ltd., Cambridge, UK) equipped with a cation exchange column (u-3183, 200 mm bed length and 4.6 mm diameter; Biochrom Ltd., Cambridge, UK). Post-column derivatization with ninhydrin was used for the detection of amino acids. The system was operated at a flow rate of 18 mL/h. The amino acid content was expressed as the number of amino acid residues per 1,000 residues.

#### 2.6.3 Thermal behaviors

Collagen samples were accurately weighed into a Hastelloy C crucible and rehydrated with 0.5 M acetic acid at 4 °C overnight. Thermal analysis was performed using a micro-differential scanning calorimeter (µDSC) (7 evo microcalorimetry; Setaram Instrumentation, Caluire-et-Cuire, France). The instrument was calibrated using a naphthalene standard prior to the analysis. The samples were heated from 20 to 80 °C at a constant heating rate of 1 °C/min. Thermal denaturation (T_d_) was expressed in °C, and enthalpy change (ΔH) was calculated in J/g collagen on a dry basis.

#### 2.6.4 Spectral characteristics

##### 2.6.4.1 Circular dichroism (CD) spectroscopy

Collagen samples were dissolved in 0.05 M acetic acid and incubated at 4 °C overnight. The supernatants were collected and diluted to obtain a protein concentration of 0.2 mg/mL using a Nanodrop spectrophotometer (Nanodrop oneC; Thermo Fisher Scientific, Madison, WI, USA). The solubilized samples (100 µL) were transferred to a V-bottomed 96-well plate. The CD profiles were recorded using a Jasco-CD spectrophotometer (J-815, Jasco International Co., Ltd., Tokyo, Japan) combined with an autosampler system (Tecan Systems, San Jose, CA, USA) and a photomultiplier (PMT) detector. CD patterns were collected between 190 and 260 nm with five accumulations at a scan speed of 100 nm/min. Measurements were performed using a quartz cell with a path length of 1 mm and a spectral bandwidth of 1 nm.

##### 2.6.4.2 Synchrotron radiation wide-angle X-ray scattering (SR-WAXS)

The crystalline and amorphous structures of collagen samples were analyzed by wide-angle X-ray scattering at beamline BL1.3 W, Synchrotron Light Research Institute (SLRI, Thailand). Approximately 200 mg of lyophilized collagen was sealed between Kapton films and mounted on a sample holder. The incident X-ray beam was operated at 9 keV with a photon flux of 2 × 10^9^ photons s^−1^. Scattering patterns were collected using a CCD detector (Rayonix LX170HS). Data were acquired over a 2θ range of 5°–45° with an exposure time of 5 min. The sample-to-detector distance and detector parameters were set according to beamline configuration. Instrument calibration was performed using 4-bromobenzoic acid as a standard.

The resulting diffraction patterns were normalized and processed using SAXSIT software (BL1.3 W, SLRI) to evaluate structural organization and intermolecular ordering of collagen.

##### 2.6.4.3 Fourier-transform infrared (FT-IR) spectroscopy

Infrared spectra were recorded using an FT-IR spectrometer (Tensor 27, Bruker, Germany) equipped with a focal plane array detector (Hyperion 3000). Lyophilized collagen samples were analyzed by attenuated total reflectance (ATR) over the range of 4,000–900 cm^−1^ at a resolution of 4 cm^−1^ with 128 scans. Spectra were processed using OPUS software (v7.5, Bruker), including water compensation, smoothing, normalization, and baseline correction. The ratio of amide III (1,300–1,200 cm^−1^) to CH_2_ bending (1,450 cm^−1^) bands was calculated to assess triple-helix integrity. The amide I region (1,700–1,600 cm^−1^) was deconvoluted using mixed Gaussian/Lorentzian functions to estimate secondary structural components, including α-helix (1,659–1,649 cm^−1^), β-sheet (1,695–1,670 cm^−1^), β-turn (1,678–1,664 cm^−1^), and random coil (1,647–1,641 cm^−1^) [28], [29]. Second-derivative spectra were generated using the Savitzky–Golay algorithm to resolve overlapping bands within the amide I–II region (1,700–1,500 cm^−1^).

#### 2.6.5 Protein identification

Collagen was identified by in-gel tryptic digestion followed by LC–MS/MS analysis, adapted from Zou et al. [11]. Protein bands excised from SDS-PAGE gels were destained with 25 mM ammonium bicarbonate in 50 % acetonitrile, reduced with 10 mM dithiothreitol (80 °C, 30 min), and alkylated with 50 mM iodoacetamide (dark, 30 min). Gel pieces were digested overnight at 37 °C with sequencing-grade trypsin (10 µg/mL). Peptides were extracted with 0.1 % formic acid, concentrated under vacuum, and reconstituted in 0.1 % formic acid containing 2 % acetonitrile.

Peptide separation was performed on a nano-UHPLC system (UltiMate™ 3000 RSLCnano, Thermo Fisher Scientific) coupled to a Q-TOF Compact II mass spectrometer (Bruker) with CaptiveSpray ionization. Samples (1 µL) were loaded onto a C18 capillary column and eluted at 300 nL/min using a linear gradient of 2–85 % mobile phase B (80 % acetonitrile, 0.08 % formic acid) over 50 min, followed by 80 % B for 10 min. Mass spectra were acquired in positive ion mode over an *m*/*z* range of 300–1,500.

Protein identification was performed by database searching against SwissProt (Metazoa) using the MASCOT search engine. Search parameters included trypsin specificity (one missed cleavage), carbamidomethylation of cysteine (fixed), oxidation of methionine and hydroxylation of proline (variable), and mass tolerances of ± 1.6 Da (precursor) and ± 0.8 Da (fragment).

Peptide identification was accepted when supported by a MASCOT ion score exceeding the significance threshold (*p* < 0.05), with a minimum of two unique peptide sequences identified per chain and at least five matched fragment ions per peptide. Protein assignments were performed at the individual collagen chain level. Collagen type was confirmed by the identification of at least two unique chain-specific diagnostic peptides for each collagen chain, each exceeding the MASCOT significance threshold (*p* < 0.05), and consistently detected across multiple independent MS/MS spectra.

### 2.7 Statistical analysis

All experiments were performed using three independent biological replicates (three separate trachea batches from three individual collection occasions), each analyzed in triplicate, resulting in n = 9 measurements per condition. For the HIU treatments, each intensity–time combination was evaluated using independently sourced biological samples. Graphs were generated using GraphPad Prism (version 10.0; GraphPad Software, San Diego, CA, USA). The data were analyzed using a completely randomized design (CRD). One-way analysis of variance (ANOVA) and Duncan’s multiple range test with a 95 % confidence level (*p* < 0.05) were performed using IBM SPSS Statistics version 24.0 (IBM Corp., Armonk, NY, USA).

## 3 Results and discussion

### 3.1 Morphology and composition of porcine trachea tissues

**Fig. 1:**
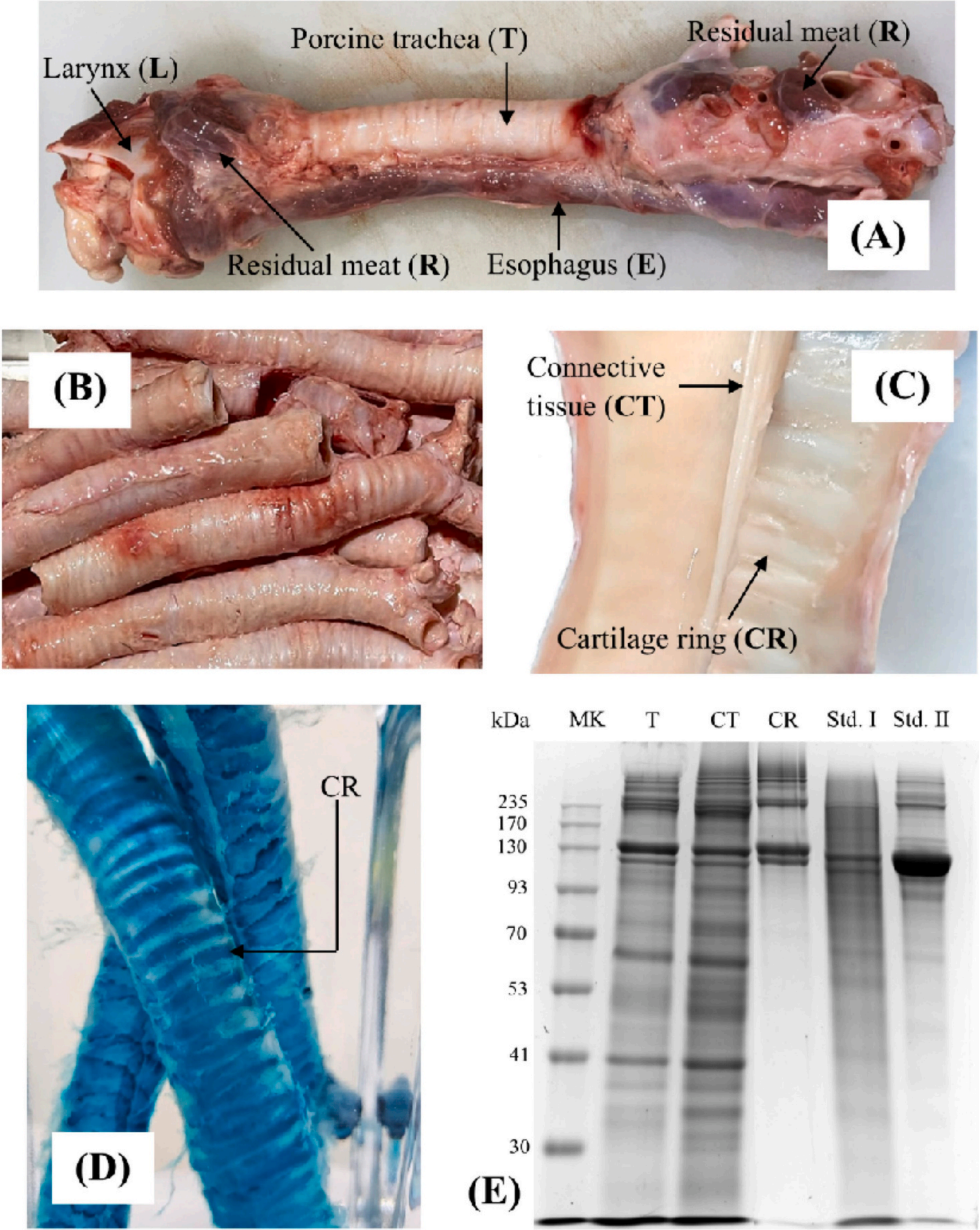
Morphological and anatomical features of porcine trachea and its derived fractions. (A) Whole porcine trachea showing anatomical association with the larynx (L), esophagus (E), and residual meat (R). (B) Bulk tracheal samples after removal from surrounding tissues. (C) Cross-sectional view illustrating separation of connective tissue (CT) and cartilage rings (CR). (D) Diaphonized trachea stained with alcian blue, highlighting the cartilage ring (CR) structure. (E) SDS-PAGE profiles of proteins from trachea (T), connective tissue (CT), and cartilage rings (CR), compared with molecular weight marker (MK) and collagen standards (type I and type II).

The macroscopic structure of porcine trachea is shown in Fig. 1A–B, where the tracheal tube is associated with adjacent tissues, including the larynx, esophagus, and residual meat. The isolated trachea exhibited a uniform tubular morphology with regularly distributed cartilage rings. Proximate analysis (Table 1) revealed that trachea is protein-rich (70.27 %, dry basis) with the highest hydroxyproline (Hyp) content, corresponding to a collagen content of 36.39 %, markedly exceeding that of surrounding tissues. In contrast, residual meat contained higher lipid levels and negligible collagen, highlighting the specificity of tracheal tissue as a collagen source [2], [30].

**Table 1:** Proximate composition of the porcine trachea and associated tissues.

**Composition**s	**Sample/ Content (%)**			
	Trachea	Esophagus	Larynx	Residual meat
1. Moisture	74.17 ± 0.71^b^	78.70 ± 1.12 ^a^	68.25 ± 1.43^c^	67.37 ± 3.54^c^
2. Ash	3.62 ± 0.16^b^	3.67 ± 0.21^b^	5.19 ± 0.52 ^a^	0.02 ± 0.00^c^
3. Total lipid	22.23 ± 1.21^c^	20.43 ± 2.97^c^	32.25 ± 3.25^b^	58.96 ± 4.07 ^a^
4. Crude protein	70.27 ± 1.17^b^	74.84 ± 0.55 ^a^	45.94 ± 2.59^c^	34.71 ± 2.24 ^d^
− Hydroxyproline (Hyp)	4.79 ± 0.03 ^a^	2.93 ± 0.52^b^	2.76 ± 0.03^b^	1.19 ± 0.06^c^
− Collagen	36.39 ± 0.20 ^a^	21.00 ± 0.22^b^	22.27 ± 0.55^b^	9.03 ± 0.45^c^
5. Carbohydrate	3.87 ± 1.82^c^	3.61 ± 3.40^c^	16.62 ± 1.82 ^a^	6.31 ± 5.12^b^

All chemical compositions are expressed on a dry basis (% dry basis; %DB), except for moisture content; Collagen content was calculated as hydroxyproline × 7.6 (conversion factor for type II collagen); Carbohydrate content was calculated by 100 − (ash + total fat + protein); Superscript letters indicate significant differences (*p* < 0.05) among values in the same row.

**Table 2:** Chemical composition and type II collagen distribution in porcine tracheal fractions.

**Composition**s	**Sample/ Content**		
	Trachea (T)	Connective tissue (CT)	Cartilage ring (CR)
Crude protein (%)	70.27 ± 1.17 ^a^	61.43 ± 1.70^b^	56.14 ± 1.04^c^
Hydroxyproline (%)	4.79 ± 0.03^b^	2.70 ± 0.33^c^	6.58 ± 1.06 ^a^
Collagen (%)	36.39 ± 0.20^b^	20.53 ± 2.52^c^	50.03 ± 1.06 ^a^
Type II collagen (µg/100 mg)	4.30 ± 0.22^b^	0.02 ± 0.00^c^	5.50 ± 0.04 ^a^

All chemical compositions were expressed on a dry basis. Superscript letters indicate significant differences (*p* < 0.05) among the values in the same row.

**Fig. 2:**
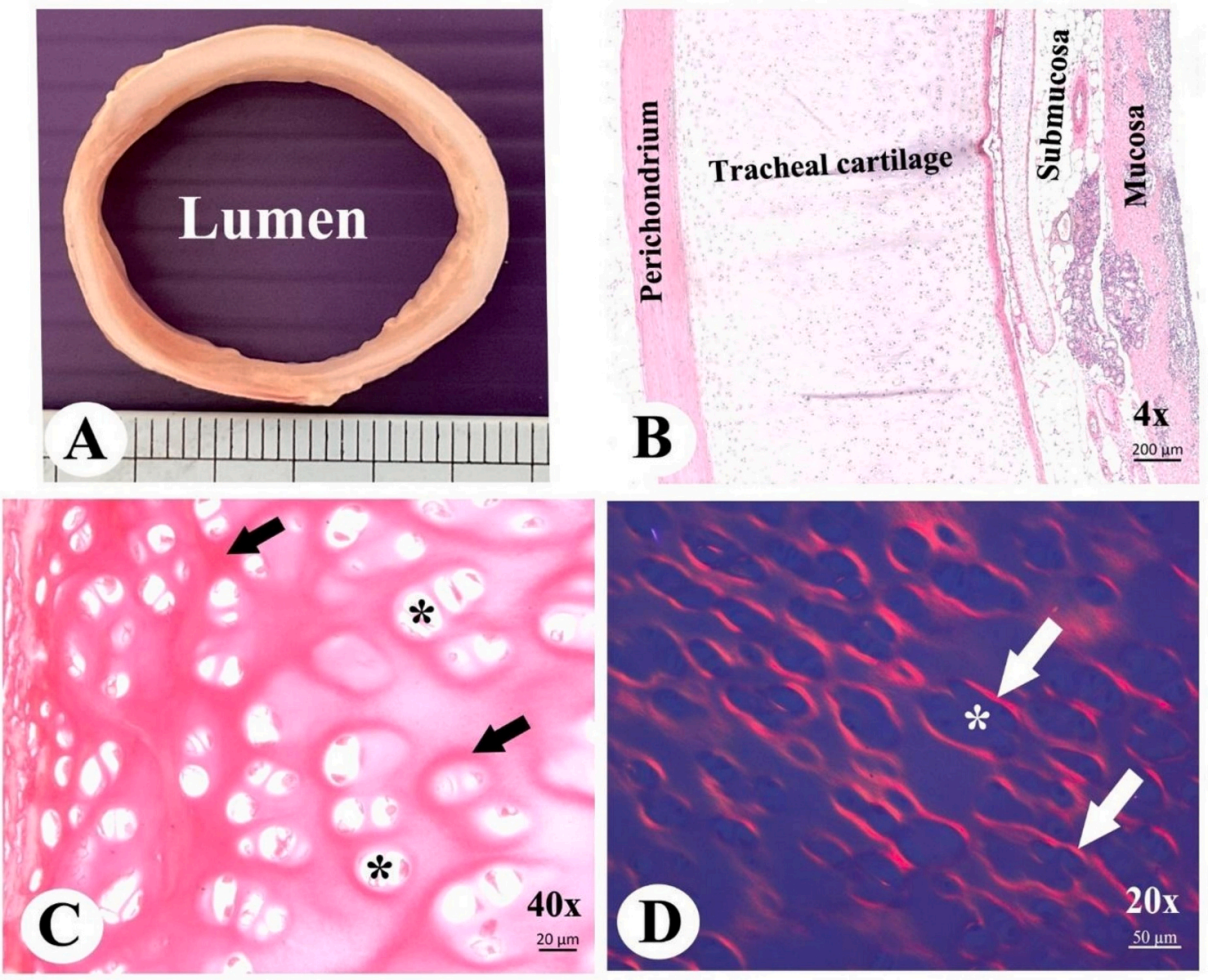
Morphological and histological organization of porcine tracheal cartilage**.** (A) Cross-sectional view of porcine trachea showing the lumen and circular cartilage ring structure. (B) Hematoxylin and eosin (H&E) staining illustrating the overall tissue architecture, including perichondrium, tracheal cartilage, submucosa, and mucosa layers. (C) Higher-magnification H&E image showing chondrocytes embedded within lacunae (asterisks) in the extracellular matrix, with surrounding territorial matrix (arrows). (D) Picro-Sirius Red staining under polarized light highlighting collagen fiber organization within the cartilage matrix (arrows), with chondrocytes indicated (asterisks). Scale bars are indicated in each panel.

Histological analysis (Fig. 2) demonstrated well-preserved tracheal architecture, with distinct mucosa, submucosa, and hyaline cartilage layers. Chondrocytes embedded within lacunae and strong Alcian blue staining confirmed the presence of a proteoglycan-rich extracellular matrix typical of cartilage. The cartilage rings exhibited significantly higher Hyp (6.58 %) and collagen content (50.03 %) than connective tissue, indicating that the cartilage ring is the primary collagen reservoir. These features are characteristic of type II collagen (CII)-rich tissues, supported by the abundance of glycosaminoglycans and organized matrix structure [8], [9].

Protein profiling further confirmed tissue specificity, with connective tissues dominated by type I collagen, whereas cartilage ring showed electrophoretic patterns consistent with CII. Quantitative analysis revealed that CII was predominantly localized in trachea and cartilage ring (4.30–5.50 µg/100 mg), with negligible levels in connective tissue. This compositional and structural specificity provides a strong foundation for extraction of type II collagen using targeted processing strategies such as ultrasound-assisted extraction [11], [19].

### 3.2 Yield of collagen extract

**Fig. 3:**
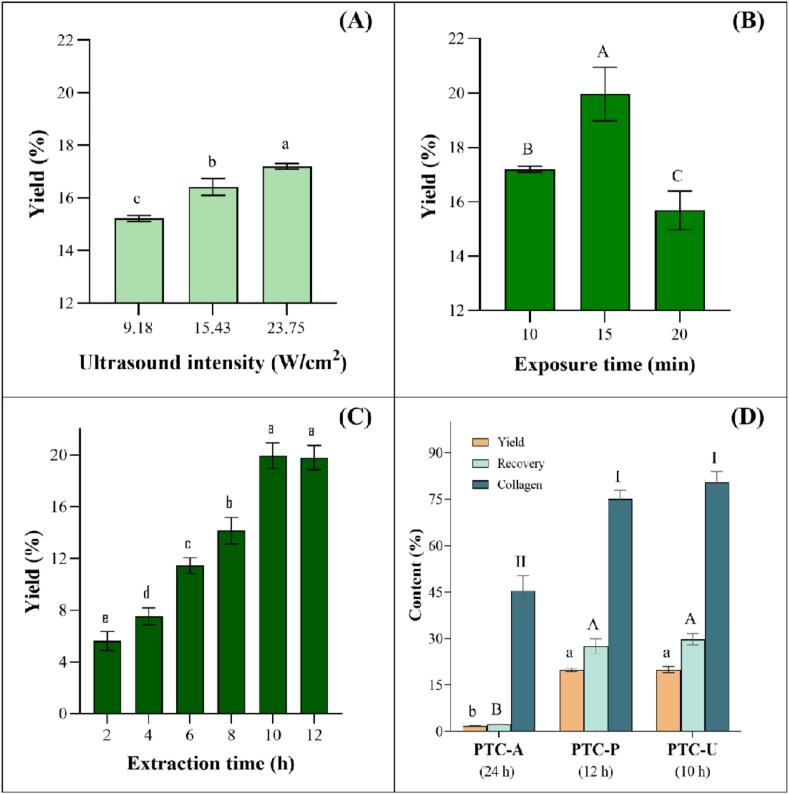
Effects of ultrasound parameters and extraction strategy on collagen yield and composition. (A) Effect of ultrasound intensity (9.18–23.75 W/cm^2^) on collagen yield at a fixed exposure time of 10 min. (B) Effect of ultrasound exposure time (10–20 min) on collagen yield at an intensity of 23.75 W/cm^2^. (C) Effect of extraction duration (2–12 h) on collagen yield under the selected ultrasound conditions. (D) Comparison of collagen yield, recovery, and collagen content obtained by acid extraction (PTC-A, 24 h), pepsin-assisted extraction (PTC-P, 12 h), and ultrasound-assisted extraction (PTC-U, 10 h). Data are expressed as mean ± standard deviation (SD, n = 9). Different lowercase letters (a–d) and uppercase letters (A–C) indicate significant differences among treatments (*p* < 0.05). Error bars in all panels represent mean ± SD.

The yield values represented total collagen content expressed as a percentage of dry weight of whole trachea. The effect of high-intensity ultrasound (HIU) on collagen extraction from porcine trachea is shown in Fig. 3. At a fixed exposure time of 10 min, collagen yield increased with ultrasound intensity from 9.18 to 23.75 W/cm^2^ (Fig. 3A), reaching a maximum at 23.75 W/cm^2^. This is likely caused by cavitation-induced microjetting and shear forces, which could further disrupt the cartilage matrix, reduce diffusional resistance, and facilitate deeper penetration of acetic acid and pepsin into the collagen network [15], [16].

At 23.75 W/cm^2^, collagen yield increased with sonication time up to 15 min but decreased at 20 min (Fig. 3B), indicating that excessive cavitation may induce partial fragmentation of collagen through localized shear and thermal effects, thereby reducing recovery of intact molecules. Because the objective of this study was to evaluate the effect of HIU processing variables on collagen extraction rather than to establish a mathematical optimum, the investigated ultrasound intensities and treatment times were selected based on preliminary experiments using a one-factor-at-a-time approach. Therefore, the selected condition (23.75 W/cm^2^, 15 min) represents the best-performing condition among those evaluated rather than the global optimum. Under the selected HIU conditions, maximum yield (19.97 %) was achieved after 10 h of extraction, with no further increase thereafter (*p* > 0.05). It should be noted that the maximum medium temperature recorded during HIU treatment (12.3 ± 1.1 °C) was well below the collagen denaturation temperature (T_d_ ≈ 39.9 °C, Table 3), confirming that enhanced yield arose from cavitation-induced mechanical disruption rather than thermal effects.

**Table 3:** Amino acid composition and thermal properties of porcine tracheal collagen extracted using different methods.

**Amino acid**	**Content (residue/ 1,000 residues)**		
	**PTC-A**	**PTC-P**	**PTC-U**
Alanine	138 ± 0.22^a^	120 ± 1.02^b^	121 ± 1.11^b^
Arginine	65 ± 0.44^b^	69 ± 0.28^a^	70 ± 0.24^a^
Aspartic/ Asparagine	28 ± 0.39	26 ± 0.10	28 ± 0.90
Glutamic/ Glutamine	93 ± 1.31^b^	97 ± 0.99^a^	98 ± 1.60^a^
Glycine	208 ± 3.29^a^	188 ± 1.92^b^	173 ± 0.13^c^
Histidine	3 ± 0.03	3 ± 0.14	2 ± 0.26
Hydroxylysine	4 ± 1.13^b^	19 ± 0.48^a^	21 ± 0.15^a^
Hydroxyproline	99 ± 1.41^b^	128 ± 1.87^a^	130 ± 2.52^a^
Isoleucine	16 ± 0.02	15 ± 0.51	15 ± 0.28
Leucine	40 ± 0.43	40 ± 0.24	41 ± 0.62
Lysine	44 ± 0.58^a^	34 ± 1.17^b^	35 ± 0.22^b^
Phenylalanine	23 ± 0.89	21 ± 0.05	22 ± 0.18
Proline	129 ± 0.12^b^	142 ± 0.70^a^	143 ± 1.82^a^
Serine	37 ± 0.62^a^	33 ± 0.95^b^	34 ± 1.15^b^
Threonine	30 ± 0.81	29 ± 0.54	32 ± 2.31
Tyrosine	2 ± 0.85	1 ± 1.05	1 ± 0.77
Valine	41 ± 0.46^a^	35 ± 0.40^b^	34 ± 0.50^b^
Imino acid (residues)	229 ± 1.28^b^	270 ± 2.57^a^	272 ± 4.34^a^
PH (%)	43.5 ± 0.37^b^	47.4 ± 0.24^a^	47.6 ± 0.17^a^
T_d_ (^o^C)	37.84 ± 0.66^b^	39.77 ± 0.30^a^	39.91 ± 0.13^a^
ΔH(J/g collagen)	0.1847 ± 0.02^b^	0.218 ± 0.01^a^	0.234 ± 0.02^a^

Imino acids represent the sum of Hyp and Pro. PH, denoting proline hydroxylation, was calculated as [Hyp / (Hyp + Pro)] × 100. Different superscripts in rows indicate significant differences (*p* < 0.05). PTC-A: porcine tracheal collagen extracted using acid, PTC-P: porcine tracheal collagen extracted using pepsin, and PTC-U: porcine tracheal collagen extracted using HIU.

Compared to conventional methods (Fig. 3D), acid extraction (PTC-A) resulted in a significantly lower yield (1.82 %, *p* < 0.05), reflecting the limited ability of acid alone to disrupt highly cross-linked cartilage matrices. Pepsin-assisted extraction (PTC-P) significantly improved yield (19.91 %) by selectively cleaving telopeptide regions, enhancing collagen solubility. However, this process remains time-intensive and dependent on matrix accessibility.

Significantly, HIU-assisted extraction (PTC-U) achieved comparable yield within a substantially shorter extraction time, demonstrating a clear advantage over conventional approaches. The improved efficiency is consistent with cavitation-driven matrix disruption and enhanced enzyme accessibility, which promote effective pepsin-mediated solubilization of cartilage-embedded collagen. Importantly, PTC-U also exhibited significantly higher collagen content (80.51 %) than PTC-P (75.04 %, *p* < 0.05), indicating more efficient recovery of collagen relative to non-collagenous components. Although HIU substantially reduced the extraction time from 24 to 10 h (approximately 58 %), the overall energy efficiency of the process at industrial scale was not evaluated. Future studies should include measurements of electrical energy consumption to determine whether the reduction in extraction time offsets the additional energy required for ultrasound treatment.

Compared to previously reported cartilage sources, including chicken sternum (5.12 %) [17], Chinese giant salamander cartilage (10.05 %) [13], and bigeye tuna bone (2.6 %) [31], the yield obtained in this study is markedly higher, highlighting porcine trachea as a highly efficient and previously underexplored source of collagen. Additionally, these conventional extractions are typically time-consuming, requiring 2–4 days for the extraction process, which limits their industrial scalability. This is the first study demonstrating that controlled HIU enables efficient extraction of collagen from porcine tracheal cartilage with simultaneous improvement in yield, purity, and processing efficiency.

### 3.3 Structural and biochemical characterization

#### 3.3.1 Microstructure

**Fig. 4:**
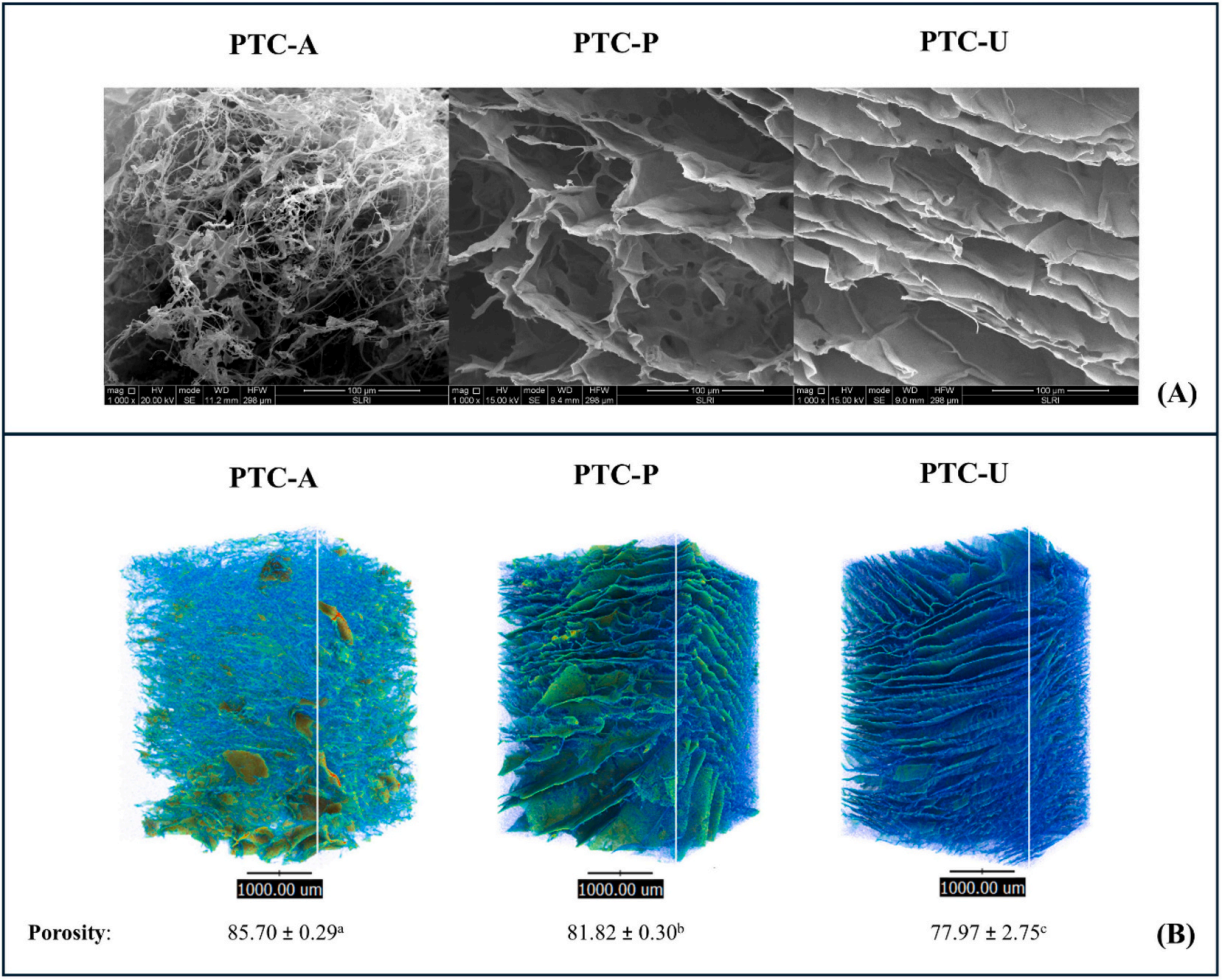
Microstructural characteristics of collagen extracted by different methods. (A) Scanning electron microscopy (SEM) images of acid-soluble collagen (PTC-A), pepsin-solubilized collagen (PTC-P), and ultrasound-assisted collagen (PTC-U) at 1,000 × magnification. (B) Three-dimensional microstructures reconstructed by synchrotron radiation X-ray tomographic microscopy (SR-XTM). Quantitative porosity values (%) are presented below each sample (mean ± SD), with different superscript letters indicating significant differences (*p* < 0.05). Scale bars: 100 μm (SEM) and 1,000 μm (SR-XTM).

SEM revealed that lyophilized PTC-A exhibited a loosely entangled fibrillar network with irregular pores (Fig. 4A), likely resulting from acid-induced swelling and partial separation of collagen fibrils under acidic conditions [10], [13]. In contrast, PTC-P displayed smoother surfaces with emerging lamellar features, reflecting improved collagen solubilization following pepsin-mediated cleavage of non-helical telopeptide regions while preserving the triple helix [10], [31]. It is noteworthy that PTC-U exhibited a markedly denser and more compact lamellar (layer-like) architecture compared to PTC-P (Fig. 4A). This architectural refinement provides molecular evidence that ultrasonic cavitation selectively destabilizes the tracheal matrix. By neutralizing the dense proteoglycan–collagen interactions through cavitation-induced shear, HIU ensures that collagen is recovered in a state that favors cohesive lamellar reorganization rather than disordered fibrillar aggregation.

These observations were further supported by SR-XTM analysis (Fig. 4B), which provided three-dimensional visualization of collagen architecture. PTC-A showed a highly porous and disordered network, whereas PTC-P exhibited partial structural organization. In contrast, PTC-U displayed a compact and stratified lamellar structure with significantly reduced porosity compared to PTC-A and PTC-P (*p* < 0.05, Fig. 4B).

As all samples were lyophilised under identical conditions (same freeze-dryer, cycle parameters, and storage at − 20 °C), the observed differences in porosity are attributed to intrinsic structural differences in the collagen networks rather than freeze-drying artefacts. The consistency between SEM and SR-XTM results confirms that HIU enhances matrix disruption and collagen extraction while promoting the formation of a dense, lamellar three-dimensional network without compromising structural integrity. This combined microstructural evidence provides direct insight into ultrasound-induced reorganization of collagen and highlights the effectiveness of HIU in extracting structurally intact collagen from porcine trachea. Such combined SEM and synchrotron-based 3D analysis of collagen structure remains limited in studies of cartilage-derived collagen.

#### 3.3.2 Amino acid profiles

The amino acid compositions of collagen extracted by different methods are presented in Table 3. Glycine was the most abundant amino acid in all samples, accounting for 173–208 residues per 1,000 residues. These values are within the reported range for type II collagen from cartilaginous tissues (170–220 residues per 1,000 residues) and are characteristic of the Gly–X–Y repeating motif that is essential for formation of the collagen triple helix [6]. Proline (Pro) and hydroxyproline (Hyp), the dominant imino acids, were also highly abundant, reflecting their critical role in stabilizing the triple-helical structure through pyrrolidine ring rigidity and hydrogen bonding [12], [31], [32].

Compared to the acid-extracted samples, PTC-P and PTC-U exhibited higher imino acid and hydroxylysine (Hyl) contents than PTC-A, indicating a greater degree of preserved triple-helical domains. This suggests that pepsin-assisted extraction, particularly when combined with HIU pretreatment, enhances solubilization of structurally intact collagen while selectively removing cross-linked telopeptide regions [10]. The imino acid patterns observed in this study are consistent with those previously reported for collagens extracted from Amur sturgeon cartilage, Chinese giant salamander cartilage, and chicken trachea [[12], [13], [21]].

The amino acid profiles of PTC-P and PTC-U closely resembled those reported for type II collagen from cartilaginous tissues, characterized by relatively high Glu/Gln and Hyl contents [12], [13], [25]. Importantly, the similarity between PTC-P and PTC-U indicates that HIU pretreatment does not alter the primary structure of collagen. These findings confirm that porcine trachea, particularly its cartilage fraction, is a rich and reliable source of type II collagen with preserved molecular composition.

#### 3.3.3 Thermal behaviors

The thermal properties of collagen samples are summarized in Table 3. PTC-P and PTC-U exhibited higher denaturation temperatures (T_d_) and enthalpy (ΔH) than PTC-A, indicating enhanced thermal stability. This improvement is closely associated with their higher imino acid content, which stabilizes the triple helix via hydrogen bonding and conformational rigidity [6], [14].

The comparable thermal properties of PTC-P and PTC-U further suggest that HIU pretreatment does not compromise collagen stability, despite significantly enhancing extraction efficiency. The T_d_ values observed in this study are higher than those reported for collagen derived from aquatic species (28.3 to 34.4 °C) [31], [33], but lower than those reported f terrestrial cartilage (43.5 to 63.0 °C) [13], [14]. This is mainly due to higher imino acid content in collagens derived from terrestrial animals, which normally contain approximately 220 to 225 residues/1,000 residues, compared to 148–206 residues in fish collagen. These results demonstrate that collagen extracted from porcine trachea retains favorable thermal stability, which is essential for maintaining functionality during processing and application [2], [33].

#### 3.3.4 Structural characteristics

##### 3.3.4.1 Circular dichroism (CD) spectroscopy

**Fig. 5:**
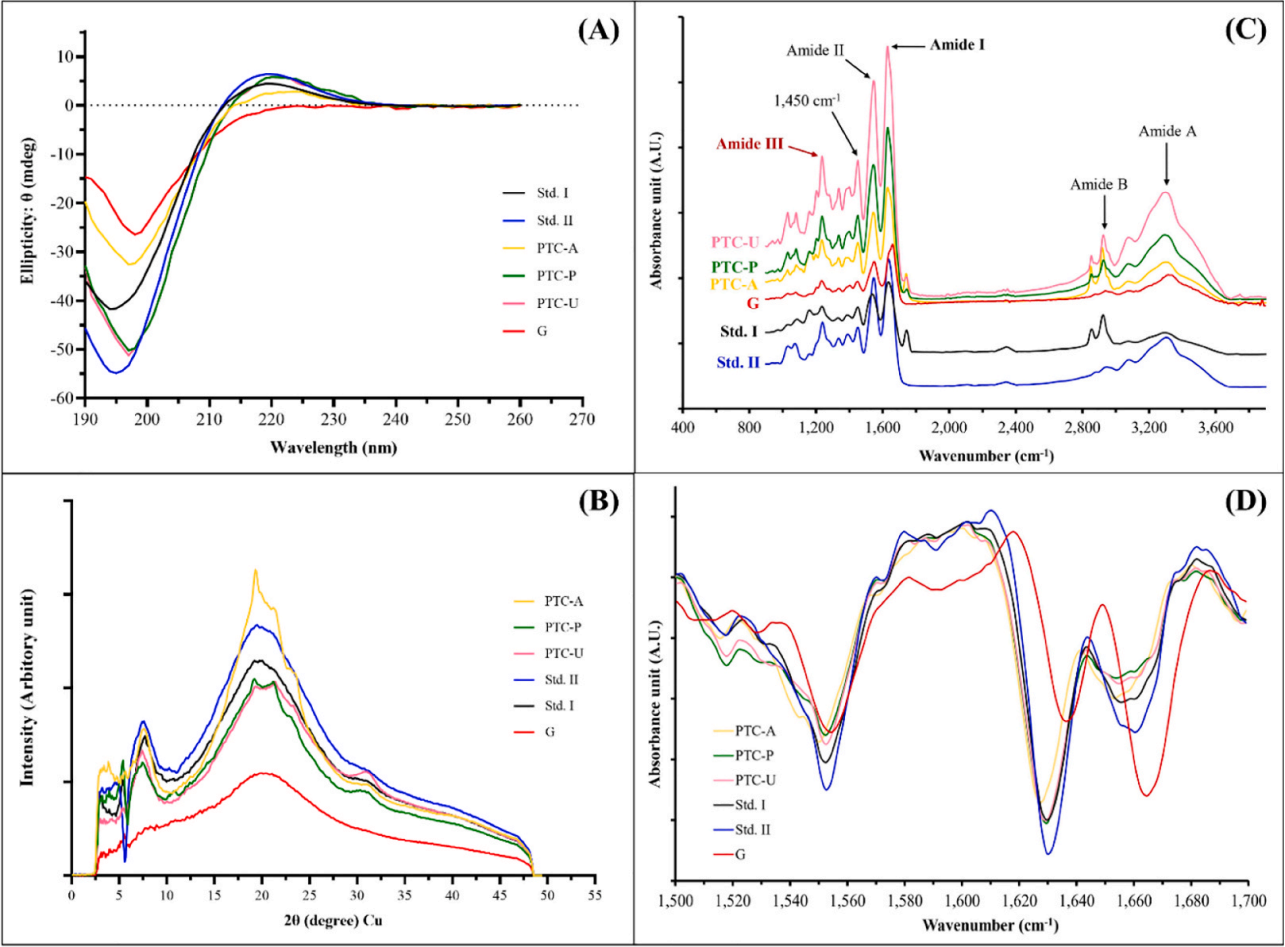
Spectroscopic characterization of collagen extracted by different methods. (A) Circular dichroism (CD) spectra of acid-soluble collagen (PTC-A), pepsin-solubilized collagen (PTC-P), ultrasound-assisted collagen (PTC-U), and collagen standard. (B) Synchrotron radiation wide-angle X-ray scattering (SR-WAXS) diffractograms over a 2θ range of 5°–45°. (C) Fourier transform infrared (FT-IR) spectra in the range of 4,000–900 cm^−1^. (D) Second-derivative FT-IR spectra of panel (C), highlighting band resolution within the amide I–II region.

CD spectra of all collagen samples exhibited a characteristic positive peak at 220 nm and a negative peak at 197 nm (Fig. 5A), confirming the presence of a native triple-helical structure [34]. The ratio of positive to negative ellipticity (RPN; Table 4) was consistent across the extracted collagen samples, ranging from 0.09 to 0.12, and was comparable to those of the type I and type II collagen standards, indicating preservation of the triple-helical structure. In contrast, gelatin exhibited only a weak, poorly defined positive band near 220 nm and a markedly lower RPN value (0.04), reflecting extensive disruption of the triple-helical conformation. Kaewbangkerd et al. [21] also reported a similar RPN for intact collagen from broiler chicken tracheas. This indicates that HIU-assisted extraction of PTC at 23.75 W/cm^2^ for 15 min did not cause a significant helix-to-coil transition. In contrast, the significantly diminished RPN value of gelatin reflects a complete conformational loss. These results confirm that HIU pretreatment under optimized conditions does not induce denaturation, unlike excessive ultrasound conditions reported previously [17].

**Table 4:** Spectroscopic parameters, secondary structure composition, and α_1_/α_2_ band intensity ratio of collagen extracted using different methods.

**Sample**	RPN*	**Absorption ratio** (Amide III/ 1,450 cm^- 1^)	**Protein secondary structure /relative content** (%)				**Protein band intensity ratio** (α1/α2 band)
			**α-helix**	**β-sheet**	**β-turn**	**random coil**	
PTC-A	0.09 ± 0.00^a^	0.95 ± 0.02^b^	27.47 ± 0.24^b^	30.22 ± 1.77^a^	18.06 ± 1.03^b^	16.86 ± 2.11^ab^	1.31 ± 0.11^c^
PTC-P	0.12 ± 0.01^a^	1.04 ± 0.03^b^	30.85 ± 1.18^a^	29.37 ± 1.00^a^	15.19 ± 1.69^c^	15.46 ± 0.65^b^	2.55 ± 0.10^b^
PTC-U	0.11 ± 0.00^a^	1.01 ± 0.03^b^	32.36 ± 1.48^a^	30.37 ± 1.61^a^	15.19 ± 1.47^c^	15.46 ± 0.82^b^	2.83 ± 0.09^b^
Std. I	0.11 ± 0.00^a^	1.23 ± 0.11^a^	32.38 ± 1.08^a^	29.87 ± 2.39^ab^	13.83 ± 3.77^bc^	16.03 ± 1.37^ab^	1.23 ± 0.04^c^
Std. II	0.12 ± 0.02^a^	1.13 ± 0.00^a^	32.80 ± 2.06^a^	27.41 ± 1.09^ab^	13.72 ± 2.60^c^	16.86 ± 1.05^ab^	3.31 ± 0.16^a^
G	0.04 ± 0.02^b^	0.72 ± 0.01^c^	21.92 ± 1.78^c^	26.48 ± 1.35^b^	33.33 ± 2.39^a^	18.24 ± 1.37^a^	ND

The Ratio of Positive to Negative peak intensity (RPN) represents the proportion of CD ellipticity between the maximum and minimum peaks. Superscript letters indicate significant differences within the same column (*p* < 0.05); PTC-A: porcine tracheal collagen extracted by acid, PTC-P: porcine tracheal collagen extracted by pepsin, PTC-U: porcine tracheal collagen extracted by HIU. I: type I collagen from calf skin; Std. II: type II collagen from chicken sternum; G: gelatin from porcine skin; ND: not determined.

##### 3.3.4.2 Synchrotron radiation wide-angle X-ray scattering (SR-WAXS)

WAXS analysis provided insight into the degree of molecular ordering and amorphous character of collagen (Fig. 5B). All lyophilized samples exhibited characteristic diffraction features associated with triple-helical packing and intermolecular spacing [35], [36]. However, PTC-A showed broader and less defined diffraction patterns, indicating a lower degree of structural organization and higher amorphous character, likely due to acid-induced swelling and fibrillar separation. Conversely, PTC-P and PTC-U displayed sharper and more distinct diffraction features, suggesting improved intermolecular packing and reduced structural disorder. The enhanced molecular alignment evidenced by WAXS correlates with the dense lamellar architecture and reduced porosity revealed by SR-XTM, suggesting that HIU-assisted extraction facilitates structural refinement across multiple scales, from sub-nanoscopic packing to macroscopic network assembly.

##### 3.3.4.3 Fourier-transform infrared (FT-IR) spectroscopy

FT-IR spectra (Fig. 5C) revealed characteristic amide bands (I, II, and III) consistent with collagen structure. The amide I band (1,630–1,635 cm^−1^) corresponds to C=O stretching, while amide II reflects N–H bending vibrations [34], [35]. The amide III/CH_2_ ratio (Table 4) observed for PTC-P (1.04) and PTC-U (1.01) confirms that the triple-helical integrity remained intact [37]. The amide III/CH_2_ bending ratio ranged from 1.01 to 1.04, remaining close to unity and consistent with preservation of the native collagen structure [29]. Although these values were slightly lower than that of the commercial type II collagen standard (1.13), they satisfy the commonly accepted criterion for intact collagen. In contrast, gelatin exhibited a ratio value of 0.72, suggesting the unfolding of triple helix. Akram and Zhang [17] reported that HIU at 11,350 W/cm^2^ for an ultrasonication time of 36 min disrupted the secondary structure of CII from chicken sternum. These findings, together with CD and WAXS results, demonstrate that HIU pretreatment effectively preserves collagen secondary structure despite enhanced extraction efficiency.

Second-derivative analysis of the amide I–II region (1,500–1,700 cm^−1^) resolved overlapping bands and clearly distinguished structural features of collagen as shown in Fig. 5D. All extracts (PTC-A, PTC-P, and PTC-U) exhibited dominant peaks at ∼ 1,650 and ∼ 1,550 cm^−1^, corresponding to amide I (C=O stretching) and amide II (N–H bending) vibrations, respectively, characteristic of intact triple-helical collagen [38]. In contrast, gelatin showed a red shift of the amide I band to ∼ 1,633 cm^−1^ with additional components (∼1,620 and ∼ 1,665 cm^−1^), indicative of helix disruption and increased random-coil content [29], [30]. Consistent with Sizeland et al. [38], the retention of the ∼ 1,650–1,654 cm^−1^ band and absence of a 1,633 cm^−1^ shift confirm preservation of the native triple helix in all extracts. These findings align with the amide III/CH_2_ ratio and amide I deconvolution (Table 4), demonstrating that HIU pretreatment (23.75 W/cm^2^, 15 min) enhances extraction without inducing structural denaturation.

#### 3.3.5 Protein patterns

**Fig. 6:**
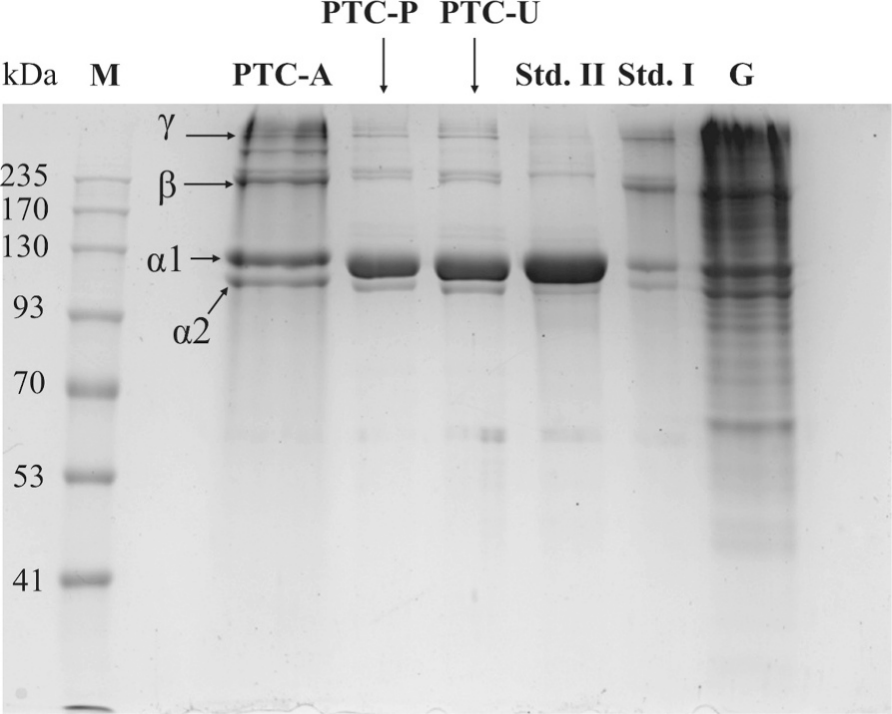
SDS-PAGE profiles of collagen extracted by different methods. Collagen samples obtained by acid extraction (PTC-A), pepsin-assisted extraction (PTC-P), and ultrasound-assisted extraction (PTC-U) are compared with molecular weight markers and reference standards. The positions of α1-, α2-, β-, and γ-chains are indicated. Std. I: type I collagen (bovine skin); Std. II: type II collagen (chicken sternum); G: gelatin (porcine skin). Molecular weight of protein markers (M) is indicated.

SDS-PAGE profiles (Fig. 6) revealed that all collagen samples contained characteristic α-, β-, and γ-chains. PTC-A displayed bands corresponding to type I collagen with α1(I) and α2(I) bands exhibiting Mw values of 129 and 118 kDa, respectively, whereas PTC-P and PTC-U were dominated by α1(II) chains, indicating enrichment of type II collagen with approximately 125 kDa. The electrophoretic profiles of CII obtained in this study corresponded well with those previously reported for collagen from porcine bone and blue shark cartilage [25], [34].

Based on the protein band intensity ratio (Table 4), the higher α1/α2 ratios observed in PTC-P (2.55 ± 0.10) and PTC-U (2.83 ± 0.09) were consistent with those of the type II collagen standard (3.31 ± 0.16), and significantly higher than the type I standard (1.23 ± 0.04, *p* < 0.05). This difference arises from the distinct chain composition of collagen types, where type I collagen consists of two α1(I) chains and one α2(I) chain [α1(I)_2_α2(I)], while type II collagen is composed predominantly of three identical α1(II) chains [α1(II)_3_]. As a result, type II collagen lacks a corresponding α2 band, leading to a markedly higher α1/α2 ratio. In this study, PTC-A exhibited both α1 and α2 chains, characteristic of type I collagen, whereas PTC-P and PTC-U showed a dominant α1 band with a reduced or negligible α2 contribution. This shift in band pattern supports the enrichment of type II collagen in these samples, in agreement with the standard CII profile.

Previous studies showed that excessive ultrasonication can adversely affect collagen structure. Chanmangkang et al. [39] reported that prolonged ultrasonication (50 % amplitude for 48 h) induced fragmentation of α1 and α2 chains in tuna tendon collagen, resulting in low-molecular-weight peptides (∼50 kDa). Similarly, Akram and Zhang [17] demonstrated that high ultrasound intensity (11,350 W/cm^2^ for 36 min) caused disorganization of α- and β-chains in type II collagen from chicken sternum. In contrast, the HIU conditions applied in this study (23.75 W/cm^2^ for 15 min) did not induce detectable polypeptide chain degradation, as evidenced by the preservation of characteristic collagen bands. This indicates that controlled ultrasound treatment can effectively disrupt the tracheal cartilage matrix without compromising molecular integrity.

### 3.4 Collagen type identification

LC–MS/MS confirmed the fibrillar collagen signature in all extracts via the conserved Gly–X–Y motif [13], [40]. As summarized in Table 5, PTC-A was identified as type I collagen based on α1(I) and α2(I) chains from *Sus scrofa*, with higher α1(I) sequence coverage (14.0 %) consistent with its 2:1 stoichiometry and preferential acid solubilization from the perichondrium. Type I–specific markers (e.g., SAGISVPGPMGPSGPR, GLPGTAGLPGMK, GEAGPQGARGSEQPQGVR) were absent in PTC-P and PTC-U, while no type II peptides were detected in PTC-A, confirming that acid extraction alone cannot effectively disrupt the dense cartilage matrix.

**Table 5:** LC-MS/MS identification of collagen chains from porcine tracheal collagen extracted using conventional and high-intensity ultrasound-assisted extractions.

**Band**	**Peptide sequence and position**	**Coverage (%)**	**Protein Identification**	**Taxonomy**
α1 PTC-A	174–189: SAGISVP**GP**M**GP**S**GP**R	14.0	Collagen α1 (I) chain	*Sus scrofa*
	256–267: GLPGTAGLPGMK			
	271–279: GFSGLDGAK			
	289–300: GEPGSPGENGAP			
	301–312: GQM**GP**RGLPGER			
	355–372: GEA**GP**QGARGSE**GP**QGVR			
	514–530: GSP**GP**A**GP**KGSPGEAGR			
	555–566: T**GP**P**GP**AGQDGR			
	577–588: GQAGVMGFP**GP**K			
	601–620: GVP**GP**PGAV**GP**AGKDGEAGA			
	621–643: Q**GP**P**GP**A**GP**AGER			
	987–996: Q**GP**S**GP**SGER			
	1,065–1,080: SGDRGET**GP**A**GP**V			
	1,081–1,095: **GP**VGAR**GP**A**GP**Q**GP**R			
	1,230–1,248DLEVDTXLKSLSQQIENIR			
α2 PTC-A	65–73: GVV**GP**QGAR	5.0	Collagen α2 (I) chain	*Sus scrofa*
	332–340: **GP**T**GP**AGVR			
	731–738: GDQ**GP**VGR			
	901–913: V**GP**R**GP**S**GP**Q**G**IR			
	887–900: GEP**GP**AGSV**GP**AGA			
	962–972: **GP**A**GP**S**GP**AGK			
α1 PTC-P	271–279: GFSGLDGAK	2.0	Collagen α1 (I) chain	*Sus scrofa*
	364–372: GSE**GP**QGVR			
	784–792: GET**GP**S**GP**A**GP**TGAR			
	1,087–1,095: **GP**A**GP**Q**GP**R			
	290–299: GYPGLDGAK	6.0	Collagen α1 (II) chain	*Sus scrofa*
	335–344: T**GP**AGAAGAR			
	384–392: **GP**EGAQ**GP**R			
	516–527: GFPGQDGLA**GP**K			
	707–718: GSPGSQGLQ**GP**R			
	1,084–1,100: QGDRGEAGAQ**GP**M**GP**AG			
	1,101–1,114: **GP**AGARGMP**GP**Q**GP**R			
	1,163–1,173 **GP**P**GP**V**GP**SGK			
α2 PTC-P	65–73: GVV**GP**QGAR	1.0	Collagen α2 (I) chain	*Sus scrofa*
	962–972: **GP**A**GP**S**GP**AGK			
α1 PTC-U	256–270: GLPGTAGLPGMKGHR	5.0	Collagen α1 (I) chain	*Sus scrofa*
	271–279: GFSGLDGAK			
	364–372: GSE**GP**QGVR			
	577–588: GQAGVMGFP**GP**K			
	987–996: Q**GP**S**GP**SGER			
	1,065–1,080: SGDRGET**GP**A**GP**A**GP**V			
	1,081–1,095: **GP**VGAR**GP**A**GP**Q**GP**R			
	335–344: T**GP**AGAAGAR	4.0	Collagen α1 (II) chain	*Sus scrofa*
	384–392: **GP**EGAQ**GP**R			
	1,084–1,105: QGDRGEAGAQ**GP**M**GP**A**GP**AGAR			
	1,163–1,173: **GP**P**GP**V**GP**SGK			
	1,231–1,248: **GP**DPLQYMRADEAAGNLR			
α2 PTC-U	85–99: GVGAGP**GP**MGLM**GP**R	2.0	Collagen α2 (I) chain	*Sus scrofa*
	157–165: GVV**GP**QGAR			
	424–432: **GP**T**GP**AGVR			

Coverage values were calculated by comparing the number of amino acid sequences identified by LC–MS/MS with the total number of amino acid residues of each collagen type. The total amino acids for each chain were as follows: collagen α1(I), α2(I), and α1(II) chains contained 1,466, 1,366, and 1,486 residues, respectively. PTC-A, PTC-P, and PTC-U are porcine tracheal collagens extracted using acid, pepsin, and HIU, respectively. Numbers before each sequence indicate the amino acid residue positions within the collagen sequence.

In contrast, PTC-P and PTC-U were identified as type II collagen through α1(II) peptides (6.0 % and 4.0 % coverage, respectively). Sequence coverage was relatively low, which is typical for fibrillar collagens. This is due to repetitive sequences and crosslinking that hinder proteolysis [13]. It is important to note that sequence coverage is not directly proportional to collagen abundance. Rather, it represents the proportion of the protein sequence covered by detected tryptic peptides and is influenced by factors such as peptide physicochemical properties, ionization efficiency, and the accessibility of cleavage sites during in-gel digestion. Therefore, the slightly lower α1(II) sequence coverage observed for PTC-U (4.0 %) compared with PTC-P (6.0 %) should not be interpreted as evidence of lower type II collagen content. Instead, it may reflect the recovery of collagen from more structurally constrained or highly crosslinked regions that are less susceptible to complete tryptic digestion. The superior performance of PTC-U is better demonstrated by its significantly higher collagen content (80.51 % vs. 75.04 %, *p* < 0.05) together with the identification of unique structural peptides derived from boundary regions. In addition, the presence of GYPGLDGAK (absent in PTC-A), which differs from the type I analogue GFSGLDGAK by a Tyr → Phe substitution, serves as a robust diagnostic marker for α1(II) [13], [25].

PTC-U uniquely yielded peptides such as QGDRGEAGAQGPMPAGPAGAR and GPDPLQYMRADEAAGNLR. Based on LC–MS/MS data (Table 5), QGDRGEAGAQGPMPAGPAGAR maps to residues 1,084–1,105 in the central triple-helical domain of the *Sus scrofa* α1(II) chain (UniProt P02458), while GPDPLQYMRADEAAGNLR maps to residues 1,231–1,248 at the C-telopeptide boundary region. The latter contains a sequence that deviates from the canonical Gly–X–Y repeating pattern, suggesting that it may originate from a structurally distinct or boundary region of the collagen chain. Their detection indicates that HIU pretreatment facilitates cavitation-induced matrix disruption, enhancing exposure of deeply embedded domains beyond the reach of pepsin alone.

Taken together, the three analytical approaches provide converging evidence for type II collagen enrichment. ELISA (Table 2) established that CII is predominantly localized in tracheal cartilage (5.50 μg/100 mg) with negligible amounts in connective tissue (0.02 μg/100 mg), providing quantitative rationale for the extraction strategy. SDS-PAGE confirmed chain-level enrichment with α1/α2 ratios in PTC-P and PTC-U approaching the type II standard (Fig. 6). LC–MS/MS further confirmed molecular identity through type II-specific peptides in PTC-P and PTC-U. The convergence of these independent lines of evidence strongly supports extraction of structurally intact CII by HIU-assisted pepsin extraction. Minor detection of α1(I) and α2(I) peptides in PTC-P and PTC-U reflects the composite structure of tracheal tissue, where cartilage interfaces with type I-rich connective regions. These proteomic findings corroborate the SDS-PAGE profiles (Fig. 6) and align with previous reports in cartilage-derived collagens from *Prionace glauca* and *Camelus bactrianus.* Collectively, the results demonstrate that extraction strategy governs both collagen type selectivity and structural accessibility, with HIU markedly enhancing extraction of type II collagen domains.

Compared with conventional type II collagen sources such as chicken sternum, bovine cartilage, or shark cartilage, porcine trachea offers practical advantages including continuous availability from commercial slaughterhouses, established collection systems, and utilization of existing by-product without requiring additional animal harvesting. These characteristics enhance its potential as a sustainable raw material for collagen recovery.

Despite advantages of HIU described above, several challenges should be considered when applying HIU for type II collagen extraction. The effectiveness of HIU depends on careful selection of processing conditions, as excessive ultrasound intensity or prolonged treatment may promote collagen fragmentation and reduce recovery, as observed after 20 min of sonication in the present study. In addition, scaling up HIU-assisted extraction may be challenging because acoustic energy distribution becomes less uniform in larger processing volumes, requiring optimization of reactor design and operating conditions. The need for specialized ultrasonic equipment also increases initial capital investment compared with conventional extraction processes. Furthermore, although HIU substantially reduced the extraction time, the overall energy efficiency and economic feasibility of large-scale implementation remain to be established through future techno-economic and life-cycle assessments.

## CRediT authorship contribution statement

**Kitsanapong Kaewbangkerd:** Writing – review & editing, Writing – original draft, Visualization, Methodology, Investigation, Formal analysis, Data curation. **Ekkasit Kanklang:** Writing – review & editing, Writing – original draft, Investigation. **Apichart Ngernsoungnern:** Writing – review & editing, Supervision, Resources, Methodology, Investigation. **Catleya Rojviriya:** Visualization, Software, Investigation. **Sukanya Tastub:** Software, Investigation. **Sittichoke Sinthusamran:** Resources, Funding acquisition. **Jirawat Yongsawatdigul:** Writing – review & editing, Supervision, Project administration, Funding acquisition, Conceptualization.

## Declaration of competing interest

The authors declare that they have no known competing financial interests or personal relationships that could have appeared to influence the work reported in this paper.
